# Brand attitude as the catalyst: transforming perceived ESG to consumer's purchase intention in low-carbon consumption

**DOI:** 10.3389/fpsyg.2025.1599472

**Published:** 2025-10-20

**Authors:** Ran Zhang, Xiaolong Zhou, Qijia Liu, Chen Wang

**Affiliations:** ^1^School of Business Administration, Shandong Women's University, Jinan, Shandong, China; ^2^Teaching Affairs Department, Shandong Polytechnic College, Jining, China; ^3^Department of Global Trade and Management, Shinhan University, Uijeongbu-si, Gyeonggi-do, Republic of Korea

**Keywords:** perceived ESG, brand identification, brand trust, green skepticism, low-carbon purchase intention

## Abstract

**Introduction:**

The purpose of this research is to examine the influence of consumers' perceptions of corporate ESG (Environmental, Social, Governance) performance on brand-related attitude and sustainable consumption behaviors, with attention to the boundary conditions imposed by varying degrees of green skepticism toward low-carbon offerings.

**Methods:**

This study conducts a multi-wave survey design to test all hypotheses.

**Results:**

The empirical study establishes three critical pathways: (1) All perceived ESG dimensions exert significant positive effects on brand trust, (2) Environmental and governance indicators emerge as unique predictors of brand identification, and (3) These attitudinal shifts, in turn, positively influence consumers' intentions to purchase low-carbon products.

**Discussion:**

Theoretical contributions are twofold: First, the integration of signaling theory with brand attitude frameworks elucidates the psychological sequencing through which institutional sustainability signals translate into purchase behaviors. Second, the typological approach to green skepticism advances existing environmental psychology models by delineating context-specific moderation effects. Practically, the results provide actionable insights for marketers aiming to design effective ESG communication strategies and foster consumers' positive brand attitude in low-carbon consumption.

## 1 Introduction

Amid escalating global ecological imperatives, low-carbon consumption has emerged as a pivotal behavioral strategy for climate mitigation (Wang et al., 2024a,b). Concurrently, ESG criteria have become central to corporate sustainable investment evaluations. However, theoretical integration of consumer perceived ESG (P-ESG) within established behavioral frameworks remains insufficiently developed, particularly regarding its influence on sustainable consumption patterns (Oh et al., 2024). Previous research inadequately addresses three gaps: (1) the cognitive encoding of P-ESG cues in consumers' brand evaluation model, (2) boundary conditions affecting ESG-behavior linkages, and (3) marketing theory's role in conducting ESG strategies. To address these research gaps, this study advances processual models examining how consumers' environmental, social, and governance perceptions differentially shape their brand attitudes (including brand identification and brand trust) through signaling theory, while investigating green skepticism's moderating effects on low-carbon purchase intentions (LCPI). These insights clarify the psychological sequencing through which institutional ESG performance translates to consumer engagement, offering marketers evidence-based strategies to align sustainability initiatives with consumption decision-making (Huang et al., 2024).

Previous studies have identified multifaceted antecedents of low-carbon consumption across macro-meso-micro levels, encompassing policy frameworks, sociocultural dynamics, organizational strategic initiatives, consumer psychographics, and technological innovations (e.g., Abdulkarem and Hou, 2021). Within corporate strategic domains, extant research examines operational mediators including carbon labeling systems (Rondoni and Grasso, 2021; Zhao et al., 2018), triple-context product cues (technological, organizational and environmental), CSR communication paradigms (Wang et al., 2022), and AI-driven customization architectures (Wang et al., 2024b). Notably, ESG performance has emerged as a focal point in sustainability governance, yet its behavioral implications in consumer decision making process remain nascent (Au et al., 2023). Despite growing recognition of ESG's institutional relevance, the mechanisms through which P-ESG influences consumption behaviors—particularly through attitudinal mediators in low-carbon contexts—constitute an underexplored frontier (Huang et al., 2024). Furthermore, ESG practices inherently function as signals through which firms convey their commitment to sustainability to external stakeholders. These observable and verifiable signals align directly with signaling theory's focus on reducing information asymmetry through observable cues (Filieri et al., 2021). Therefore, this study addresses this gap by applying signaling theory to examine how P-ESG dimensions differentially (1) engender brand identification and trust, and (2) subsequently drive low-carbon product purchase intention, thereby advancing micro-level explanations of ESG's behavioral efficacy beyond current macro-oriented organizational analyses.

In the same vein, consumers' brand evaluation (including, identification and trust) refers to the subjective experience of cognitive-emotional convergence associated with completing an information-processing task (Oppenheimer, 2008). According to the signaling theory, previous research on consumer psychology and other fields has investigated that higher brand evaluation enhance individual's consumption confidence, leading to increased consumption frequency and satisfaction (Gurler, 2025; Yoshida et al., 2021). The P-ESG offers a novel approach to consumer relationship management strategy, while also delivering more comprehensive perception experiences. Therefore, investigating the mechanisms underlying the relationship between P-ESG and LCPI from the perspective of consumer's brand evaluation is crucial (Gurler, 2025). To fill this gap, this study aimed to propose the significant mediating effect of brand evaluation on the relationship between P_ESG and consumers' LCPI from two dimensions: identification and brand trust (Konuk, 2023; Yu et al., 2025). Moreover, this study further explores how consolidated brand evaluations (including brand identification and brand trust) drive consumers' LCPI. While existing research has identified key predictors of sustainable consumption—including value perceptions (Xiao et al., 2019), cognitive processing fluency (Wang et al., 2024a), and retail trustworthiness (Cui et al., 2019), critical gaps persist in understanding dual-attitude mechanisms. Recently, most models examine brand-related constructs in isolation, neglecting their synergistic effects in environmentally consequential decision-making (Yadav et al., 2024).

Furthermore, building upon Wang C. et al. (2021)'s identification of consumer bias as an understudied factor in low-carbon research, this study investigates how green skepticism—defined as pre-consumption cognitive biases toward eco-claims (Wang et al., 2024b)—moderates P-ESG driven brand attitude formation. While existing studies emphasize skepticism's role in shaping communication strategies (Luan et al., 2023) and sustainable decision-making (Wang et al., 2024b), critical gaps persist regarding its asymmetric moderating effects on brand identification (self-concept alignment) vs. brand trust (competence attribution). We extend signaling theory by examining boundary conditions under which skepticism alters P-ESG signal interpretation. This dual-mechanism analysis clarifies how preexisting biases reconfigure the attitude-intention pathway, offering empirical evidence to optimize ESG disclosure prioritization in high-skepticism markets. The findings advance micro-level explanations of green skepticism's contextual contingencies in bridging institutional ESG performance with consumer psychology.

Overall, this research makes three contributions to corporate's ESG strategy and consumers' low-carbon consumption area. First, according to signaling theory, we explain the influence mechanism of P-ESG's three dimension (environmental, social, governance) on improving consumers' brand identification and trust in complex low-carbon products consumption process. Specifically, we investigate how consumers' P-ESG for a corporate can be used to improve positive brand image and eliminate uncertainty risk, and they can finally trigger consumers' purchase intention for low-carbon market offerings. Second, we empirically investigate the relationships between consumers' brand identification, brand trust and their LCPI in a complex sustainable consumption process. Third, based on different bias and information processing mechanisms, this study investigates the moderating role of consumers' different green skepticism levels on their different information processing modes for low-carbon products.

## 2 Theoretical background and hypotheses

### 2.1 ESG effects in consumer relations

ESG originated from corporate social relationship (CSR) frameworks conceptualized in the 1960s, emphasizing economic compliance, legal adherence, ethical commitments, and philanthropic dimensions in corporate-stakeholder relations (Carroll, 1991). Research findings validate that robust ESG implementation enhances financial resilience, as socially-conscious investors increasingly prioritize comprehensive evaluations of corporate sustainability profiles (Friede et al., 2015). Current ESG research predominantly examines institutional policy-making and capital allocation in sustainable ventures, yet insufficiently addresses how corporate ESG strategies influence public perception and consumer engagement (Oh et al., 2024). Considering consumers constitute pivotal organizational stakeholders, corporations must ensure their ESG initiatives align with public expectations to optimize economic returns and foster sustainable growth through stakeholder resonance (Bridoux and Stoelhorst, 2022).

Current research about market implications of organizational ESG initiatives remain underdeveloped, while stakeholder engagement studies predominantly concentrate on sustainability management at institutional levels (Harrison, 2021). Incorporating ESG frameworks into marketing science enables corporations to synchronize their communication strategies with evolving consumer demands for enterprise-level ESG implementation (Chon and Kim, 2021). Emerging research has initiated explorations of ESG-consumer relationship dynamics, developing comprehensive metrics to quantify consumers' evaluations of corporate environmental stewardship, social accountability, and governance transparency (Oh et al., 2024). Empirical evidence confirms the substantial impact of environmental and social initiatives on advertising receptivity, brand allegiance, and purchasing patterns, whereas governance-related communication efficacy remains underexplored (Wu and Wang, 2014; Wang et al., 2024b). This research gap underscores the imperative to examine how consumer perceptions of corporate's ESG performance interact with individual pro-environmental decision-making through dual lenses of sustainable consumption patterns and micro-level behavioral analysis.

### 2.2 Relation between P-ESG and brand identification and brand trust

Grounded in established theoretical foundations, this study synthesizes firm's ESG frameworks with sustainable consumption patterns, specifically examining low-carbon product promotion. Distinct from conventional products, sustainable offerings embed ecological preservation and emission reduction principles across production cycles, supply chain operations, and consumption phases (Yu et al., 2025). When consumers perceive authentic corporate investments in emission reduction, energy optimization, and social welfare initiatives, they undergo two psychological stages—cognitive processing and emotional resonance—internalizing ESG commitments as part of their own value system. This intrinsic alignment suggests consumers' evaluations of organizational ESG commitments amplify product resonance through enhanced environmental empathy, thereby expanding the scope and intensity of eco-conscious consumption behaviors (Khalil and Khalil, 2022). Thus, this study systematically examines tripartite P-ESG dimensions—environmental consciousness, social responsibility cognition, and governance transparency perception—as critical antecedents shaping brand affiliation dynamics and trust formation mechanisms within green market contexts.

(1) The perceived environmental encapsulates consumer evaluations of organizational initiatives in ecological conservation and resource optimization (Wang et al., 2023). This construct incorporates measurable operational parameters including carbon footprint mitigation strategies, sustainable energy utilization, and circular economy adoption (Oh et al., 2024). This study operationalizes signaling theory within sustainable consumption research paradigms to decode interaction mechanisms between P-ESG constructs and brand affiliation dynamics (including brand identification and brand trust). Originating from economic sociology, signaling theory addresses knowledge disparities where demand-side stakeholders face informational disadvantages relative to supply-side entities retaining product quality superiority (Spence, 2002). Within such knowledge gaps, P-ESG cues as observable markers that decode latent brand equity components—specifically brand-customer congruence and reliability assessments - through empirically verifiable sustainability signals (Filieri et al., 2021). This theoretical framework proves particularly salient in eco-conscious market contexts where product sustainability claims require empirical substantiation.

Amid intensifying ecological crises, firm's demonstrations of decarbonization initiatives and clean production systems function as strategic differences that cultivate stakeholder alignment (Block et al., 2024). When firms operationalize sustainability commitments through verifiable emission reduction protocols and energy conservation systems, these practices emit verifiable signals that strengthen brand-customer value congruence while enhancing perceived organizational integrity (Chon and Kim, 2021). Signaling theory indicates this phenomenon through neo-institutional lenses, positing that environmental stewardship constitutes a salient value proposition for modern consumers, with such firm activities transmitting institutional legitimacy that enhances brand allegiance through sustainability credibility (Konuk, 2023). Concurrently, decarbonization implementation necessitates technological sophistication and R&D investments, which inherently signal product excellence and process innovation capabilities—critical determinants of brand identification and trust formation (Miao et al., 2020).

(2) The perceived social construct captures stakeholder evaluations of organizational commitments to value co-creation across three operational vectors: labor equity safeguards, community empowerment initiatives, and ethical operational protocols (Oh et al., 2024). Previous empirical research reveals that corporate social responsibility (CSR) perceptions significantly mediate consumer's responses to firm's communication message and brand equity formation (Chon and Kim, 2021). This multifaceted construct encompasses consumer recognition of workforce development programs, social impact investments, and human rights-compliant supply chains, which collectively drive consumption preference patterns (Agu et al., 2024). Current research prioritizes decoding perceived social signaling mechanisms within sustainable consumption paradigms, particularly examining how firm's social communications activate neural engagement pathways that convert consumers' ethical awareness into their purchase intentions (Roh et al., 2022). In this vain, some research frameworks extend beyond macro-level social responsibility - performance correlations to investigate microcosmic impacts. Specifically, how social perceptions shape consumers' trust architectures and catalyze pro-environmental consumption cycles (Konuk, 2023).

Consumers systematically attribute elevated ethical standing to firms demonstrating robust social responsibility commitments, fostering credibility that positively influences brand confidence metrics (Wang et al., 2022). This stakeholder reciprocity dynamic manifests through organizational behaviors like workforce empowerment initiatives - exemplified by professional competency enhancement programs - which generate positive externalities that elevate brand attractiveness indices and strengthen consumers' brand identification (Yoshida et al., 2021). At the same time, the formation of consumers' brand trust operates through an ethical-commercial halo effect, wherein altruistic organizational behaviors are cognitively associated with product excellence expectations (Konuk, 2023). It explains how consumers cognitively map employee-centric operational philosophies onto firm's R&D investment patterns, thereby amplifying perceived brand authenticity and brand trust potential through symbolic quality signaling.

(3) Perceived governance encompasses consumers' evaluations of corporate governance structures and practices, including dimensions such as management transparency, decision-making accountability, and ethical leadership standards (Au et al., 2023). This dimension serves as a critical system effectiveness indicator within stakeholder theory, measuring institutional alignment with rigorous compliance benchmarks and managerial integrity metrics (Oh et al., 2024). Empirical evidence demonstrates that robust governance mechanisms (including formalized board oversight procedures, accountable decision-making processes, and regulatory compliance systems) serve as verifiable indicators of operational reliability (Yu et al., 2025). Drawing on signaling theory, such governance practices signal firm commitments to sustainable development, thereby improving consumers' brand attitudes through demonstrated institutional responsibility (Konuk, 2023).

Moreover, building on signaling theory foundations, firm's governance initiatives function as tangible demonstrations of sustainable development commitments, creating positive brand attitude formation through institutional credibility (Konuk, 2023). Effective governance systems establish multilayered quality assurance protocols that reinforce product-brand congruence, particularly through standardized safety inspections and production monitoring mechanisms (Silas et al., 2022). These operational safeguards enable consumers to associate robust governance frameworks with reliable product performance indicators, thereby strengthening brand identification and trust through verifiable quality management evidence (Stokburger-Sauer et al., 2012).

In conclusion, amid intensifying climate change—marked by extreme weather and rising greenhouse gases—low-carbon development has become a global consensus and public discourse focus. This macro context elevates policy and industrial standards while reshaping consumer mental models (Waters et al., 2025). Micro-level environmental awareness accumulation prompts individuals to internalize corporate ESG practices as personal values. Perceiving genuine corporate investments in emission reduction, energy optimization, or social welfare, consumers undergo a two-stage cognition-emotion sequence: information filtering precedes positive anticipation. Sustainability signals first enhance consumers' brand evaluation; emotional resonance then strengthens brand affinity and loyalty (Konuk, 2023). These dual mechanisms collectively amplify brand attitude, catalyzing LCPI. Thus, considering the above three dimensions of P-ESG, all hypotheses are proposed as follows:

**Hypotheses:** Consumers' P-ESG (**H1a**: perceived environmental, **H2a**: perceived social, **H3a**: perceived governance) have positive effects on their brand identification.
**Hypotheses:** Consumers' P-ESG (**H1b**: perceived environmental, **H2b**: perceived social, **H3b**: perceived governance) have positive effects on their brand trust.

### 2.3 Consumers' brand identification and brand trust → LCPI relationship

Brand identification embodies a cognitive-emotional convergence where consumers assimilate brand ethos into their self-schema, generating durable emotional attachments that transcend transactional relationships (Gurler, 2025; So et al., 2017). This internalization process manifests commercially through two behavioral pathways: (1) recurrent purchasing patterns reflecting personal value actualization, and (2) spontaneous brand advocacy serving as social identity signaling (Popp and Woratschek, 2017; Yoshida et al., 2021). Firm's brand identification frameworks clarify how consumers internalize corporate sustainability agendas (including low-carbon initiatives) as extensions of personal environmental ethics, transforming eco-conscious consumption into both ideological expression and relational investment (Silas et al., 2022; Tuškej and Podnar, 2018). Unlike situational brand preferences susceptible to market fluctuations, identification-based loyalty roots consumer decisions in stable sociocultural paradigms, creating consumption patterns resilient to competing offers (Gurler, 2025). Consequently, strongly identified consumers approach low-carbon purchases as ritualistic reaffirmations of their core values rather than conventional marketplace exchanges (Jiang et al., 2020).

In the same vein, brand trust (as a consumer's confidence in a brand's integrity and competence) serves as a pivotal driver of eco-conscious purchasing decisions (Hur et al., 2014). Many empirical evidence confirms that this trust directly strengthens willingness to adopt sustainable behaviors, including participation in environmental initiatives and selection of low-carbon products (Abu Zayyad et al., 2021). When consumers perceive brands as reliable stewards of ecological responsibility, they are more likely to accept environmental claims about product benefits, in return amplifying both purchase intention and actual sustainable consumption behavior (ElHaffar et al., 2020; Hur et al., 2014). This trust-behavior linkage arises because credible brands reduce perceived risks, transforming skepticism about green claims into actionable buying commitments (ElHaffar et al., 2020).

In conclusion, compared with ordinary products, low-carbon products inherently contain a greater array of attributes pertaining to environmental protection and sustainable development (Boufounou et al., 2023; Xue et al., 2022). Whereas, they are also associated with a higher incidence of negative information (e.g., high premium, poor product quality, long delivery time, etc.). Therefore, consumers place a heightened emphasis on the identification and trust in brand, which is cultivated over an extended period and requires considerable investment, when formulating their final purchase-decisions (ElHaffar et al., 2020; Wang et al., 2024a). Based on these premises, we advance the following hypotheses:

**Hypotheses:** Consumers' brand identification (**H4**) and brand trust (**H5**) have positive effect on their LCPI.

### 2.4 The moderating effect of green skepticism

Existing research on sustainable consumption patterns has predominantly emphasized promotive psychological drivers - such as ecological consciousness and ethical self-concept, while marginalizing inhibitory cognitive-emotional variables in behavioral frameworks (Lin and Chen, 2016; Yu, 2020). Grounded in the Theory of Planned Behavior (TPB), attitudinal orientation is operationalized as consumers' evaluative judgments regarding the desirability of specific actions (Ajzen, 1991), a construct empirically validated to mediate the intention-behavior linkage in eco-conscious contexts (Chen, 2020; Tan et al., 2017). Within green consumption paradigms, product sustainability credentials critically inform attitudinal development through perceived environmental efficacy (Orazi and Chan, 2020). Nevertheless, negative forces like consumer skepticism toward ecological claims (representative yet underexamined facets of attitudinal resistance) warrant rigorous scholarly investigation to address persistent intention-behavior gap (Wu and Yang, 2018). Following this discussion, we took consumers' negative perceptions (green skepticism) into the TPB model to investigate the moderating effects of green skepticism on the relationship between P-ESG and LCPI.

The Theory of Planned Behavior (TPB) posits that behavioral intentions emerge from tripartite determinants: attitudinal evaluations, subjective norms, and perceived behavioral control (Ajzen, 1991). Subjective norms operationalize individuals' perceptions of social expectations and personal motivations regarding specific actions. Notably, inhibitory normative beliefs—particularly green skepticism—can substantially diminish eco-conscious consumption intentions (Pristl et al., 2021). Green skepticism, defined as consumer skepticism toward ecological claims of sustainability-oriented offerings, triggers compensatory information-seeking behaviors that alter decision-making calculus (Leonidou and Skarmeas, 2017). Green skepticism fundamentally alters how consumers interpret ESG-related signals through cognitive filtering and attribution Bias. More specifically, consumers with low- level skepticism engage in heuristic processing (Chaiken, 1980), accepting ESG signals at face value due to lower perceived information ambiguity. They attribute corporate ESG efforts to intrinsic values (e.g., responsibility), facilitating self-brand congruence (for consumers' brand identification) and competence inference (for consumers' brand trust). On the contrary, High-skepticism consumers adopt systematic processing (Petty and Cacioppo, 1986), scrutinizing ESG signals for consistency and credibility. They exhibit extrinsic attribution bias, interpreting ESG claims as impression management tactics (e.g., greenwashing) rather than authentic commitments (Walker and Wan, 2012). This devalues signals meant to foster self-concept alignment (brand identification) and reliability perceptions (brand trust). Moreover, this cognitive recalibration process manifests through systematic verification of green product attributes, often overriding initial perceptual evaluations (Luo et al., 2020). When juxtaposed with intrinsic motivators like P-ESG, such skepticism functions as a cognitive gatekeeper that attenuates sustainability perception-behavior linkages (Yu, 2020). Consequently, green skepticism may suppress P-ESG's efficacy while constraining LCPI through dual mechanisms of cognitive discounting and behavioral hesitation.

Consequently, this paper posits that consumers with low-level (vs. high-level) green skepticism toward product are more likely to be influenced by P-ESG all dimensions (including perceived environmental, perceived social, perceived governance). That is, for low-level green skepticism consumers, P-ESG may lead to their stronger brand identification and trust, as shown in the following hypothesis:

**H6:** Green skepticism negatively moderates the relation between P-ESG → brand identification and P-ESG → brand trust relationships; that is, consumers' high-level green skepticism (vs. low-level) reduces the positive effect of P-ESG on brand identification and brand trust.

**Figure 1:**
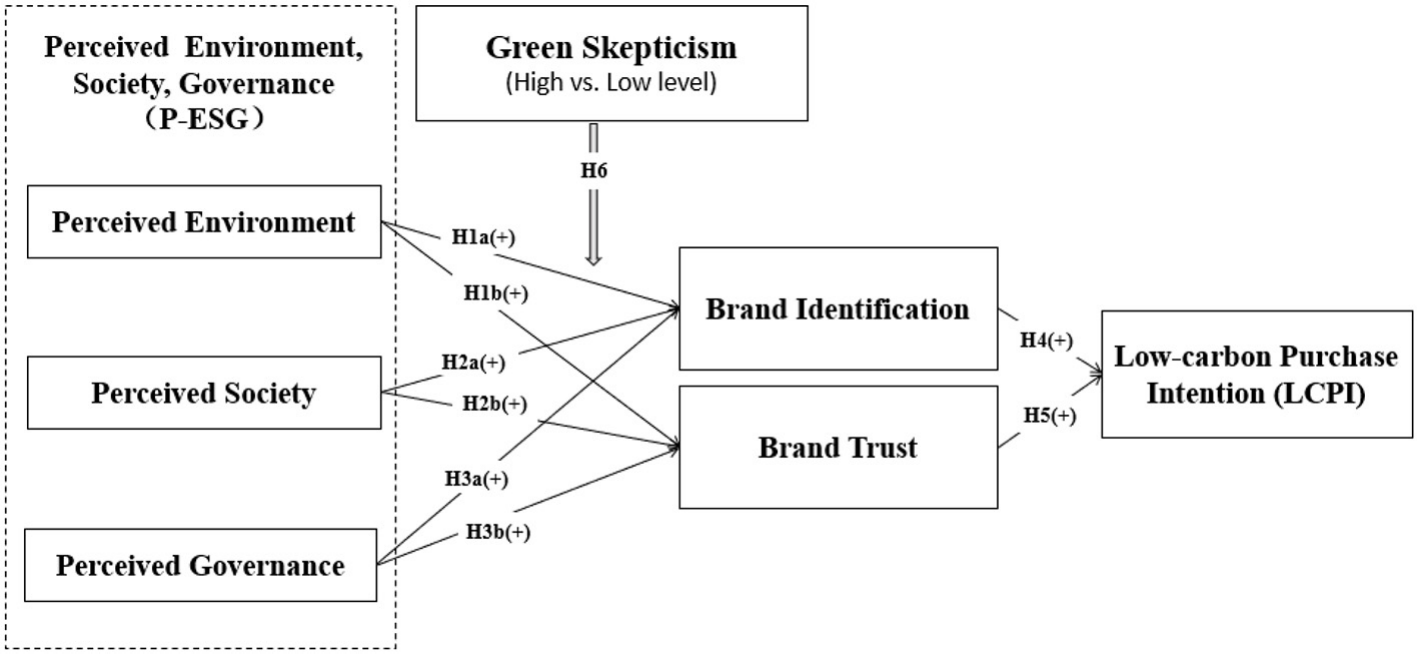
Structural model. Sources: figure by authors.

In sum, to examine the hypotheses about “P-ESG (including perceived environmental, perceived social, perceived governance) → brand identification and brand trust → LCPI” relationships between high-level (vs. low-level) consumers, we draw a structural model shown in Figure 1.

## 3 Method

### 3.1 Measurements development

Aligned with our structural model hypotheses, we employed validated multi-item scales to operationalize key constructs (see Appendix). (1) *Consumer Perceptions* (perceived environmental, perceived social, perceived governance) were each assessed through three items adapted from Oh et al. (2024), forming a nine-item composite measure. (2) *Brand Identification* was measured using a three-item scale from Tuškej et al. (2013), while *Brand Trust* utilized three items from Hur et al. (2014). (3) *Low-Carbon Purchase Intention (LCPI)* was operationalized via three indicators adapted from Wang et al. (2023). Moreover, based on foundational previous ESG communication literature (Gillan et al., 2021; Wang et al., 2024b), demographic controls included binary gender, categorical age, education level, and continuous monthly disposable income measures. All items employed 7-point Likert scales (1 = Strongly Disagree to 7 = Strongly Agree) to ensure metric consistency.

### 3.2 Survey design and sample

Following established cross-cultural research protocols (Hair et al., 2011), the English questionnaire was translated into Standard Mandarin by a team of four bilingual researchers, with independent back-translation conducted by two professors to ensure semantic consistency.

The survey was administered over eight weeks using a random-sampling design through online (WJX.com) and offline (campus and community) channels, targeted Chinese consumers with a basic understanding of ESG-related knowledge and prior low-carbon product purchasing experience (verified via screening questions). In the introductory section of the survey, we presented information on ESG performance from prominent Chinese corporations (e.g., Alibaba, JD.com, Tencent) to prime participants' awareness and retrieval of ESG-related knowledge. And then all participants were given the survey and asked to recall a company they know well, and their recent shopping experiences. Of the 480 randomly distributed questionnaires, 375 were returned (78.1% response rate), with 321 valid responses retained after excluding incomplete or invalid entries. After excluding uncompleted and invalid questionnaires, 321 sample data were used in final empirical analysis. All detailed statistics for 321 participants was shown in Table 1.

**Table 1:** Descriptive statistics of all respondent characteristics.

**Demographics**	**Category**	**Count**	**Rate (%)**
Gender	Female	164	51.1
	Male	157	48.9
Age (years)	18–25	41	12.8
	26–30	64	19.9
	31–40	164	51.1
	41–50	47	14.6
	51 or above	5	1.6
Education	Junior college	55	17.1
	Undergraduate	231	72.0
	Graduate course or above	35	10.9
Monthly disposable income (CNY)	≤ 2,500	15	4.7
	2,501–4,500	69	21.5
	4,501–6,500	127	39.6
	6,501–8,500	71	22.1
	8,501–10,500	22	6.9
	≥10,501	17	5.3
Total		321	100

Sources: table by authors.

## 4 Results

### 4.1 Non-response and common method bias

To mitigate common method variance (CMV), this study segregated independent and dependent variables across survey sections, and also controlled the time for answering each part of the questions (Podsakoff et al., 2003), and performed Harman's single-factor test via exploratory factor analysis (EFA). The EFA identified six factors with eigenvalues exceeding 1.0, collectively accounting for 76.7% of total variance, confirming negligible CMV concerns. Additionally, we addressed non-response bias through Armstrong and Overton's (1977) temporal split-sample method: Randomly dividing the sample into two groups revealed no statistically significant demographic or attitudinal differences (*p* > 0.05), validating response representativeness. Moreover, marker variables (e.g. gender, age, education and monthly disposable income) exhibited no significant impact on the model.

### 4.2 Validity and reliability of measurement model

We conducted confirmatory factor analysis (CFA) using AMOS 16 on the full sample (*N* = 321) to evaluate measurement validity across six constructs (18 items). The model demonstrated an overall good fit [χ^2^= 205.443 (*df* = 120, *p* < 0.05); *RMSEA* = 0.048; *RMR* = 0.022; *NFI* = 0.941; *GFI* = 0.937; *CFI* = 0.974], with all factor loadings exceeding 0.60 (*t* > 1.96) (see Table 2), confirming adequate convergent validity. Average variance extracted (AVE) values (0.61–0.73) surpassed the 0.50 threshold, while construct correlations remained below the square root of respective AVEs, satisfying Fornell and Larcker's (1981) discriminant validity criteria (Hair et al., 2011) (see Table 3).

**Table 2:** CFA analysis result (*N* = 321).

**Factor**	**Scale**	**S. Estimate**	***t***	**AVE**	**C.R**.
Perceived Environmental (PE)	PE1	0.848	17.386	0.726	0.888
	PE2	0.887	18.275		
	PE3	0.820	–		
Perceived Society (PS)	PS1	0.820	15.764	0.695	0.873
	PS2	0.874	16.645		
	PS3	0.807	–		
Perceived Governance (PG)	PG1	0.788	15.641	0.694	0.872
	PG 2	0.874	17.401		
	PG 3	0.835	–		
Brand Identification (BI)	BI1	0.788	14.219	0.646	0.846
	BI2	0.820	14.714		
	BI3	0.803	–		
Brand Trust (BT)	BT1	0.828	13.378	0.631	0.837
	BT2	0.830	13.392		
	BT3	0.721	–		
Low-carbon Purchase Intention (LCPI)	LCPI1	0.837	14.678	0.660	0.853
	LCPI2	0.823	14.505		
	LCPI3	0.775	–		

Model fit summary: χ2(120) = 205.443; χ2/df = 1.726; RMSEA = 0.048; RMR = 0.022; GFI = 0.937; CFI = 0.974; NFI = 0.941. Sources: table by authors.

**Table 3:** Discriminant analysis result (*N* = 321).

**Variables**	**PE**	**PS**	**PG**	**BI**	**BT**	**LCPI**
PE	0.852					
PS	0.528^**^	0.834				
PG	0.536^**^	0.385^**^	0.833			
BI	0.401^**^	0.346^**^	0.448^**^	0.804		
BT	0.567^**^	0.481^**^	0.482^**^	0.424^***^	0.794	
LCPI	0.426^**^	0.361^**^	0.302^**^	0.497^**^	0.444^**^	0.812

Diagonal value is square root of AVE value; Table value is correlation coefficient; ^**^p < 0.01; ^***^p < 0.001. Sources: table by authors.

### 4.3 Structural model

**Figure 2:**
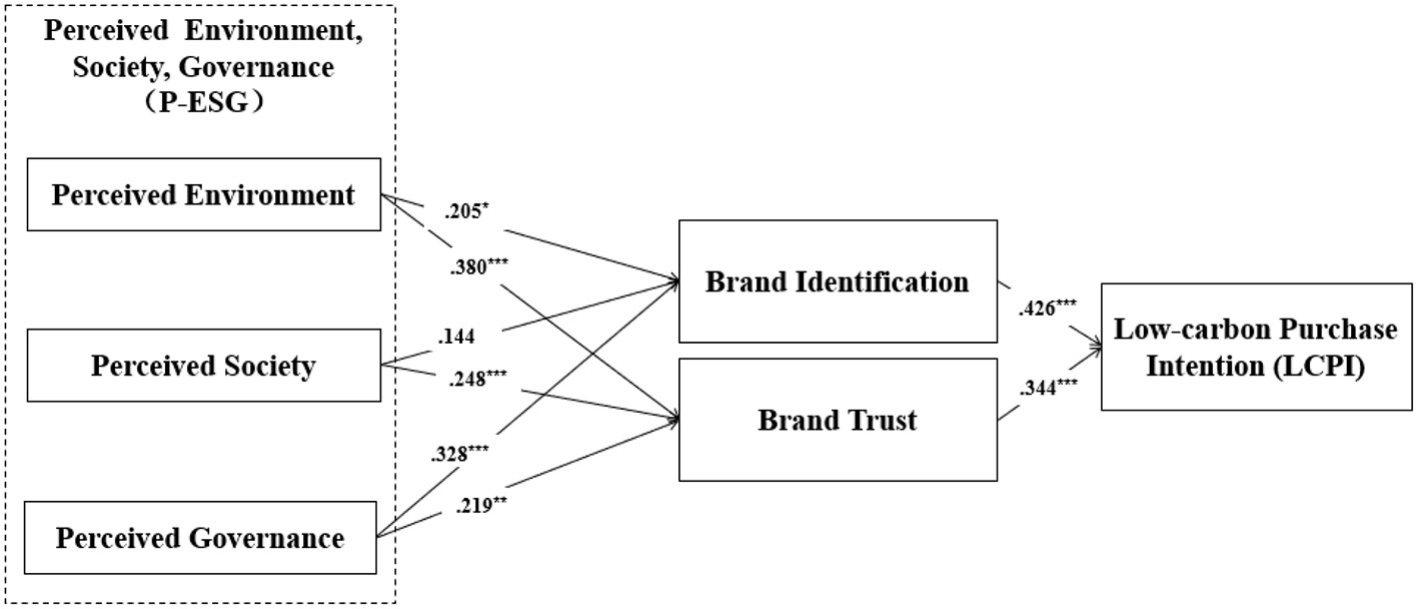
Results of structural analysis (*p < 0.05; **p < 0.01; ***p < 0.001). Sources: figure by authors.

In this study, the structural equation model (SEM) evaluated the hypothesized relationships among six constructs (three P-ESG dimensions, brand identification, brand trust, and LCPI) using AMOS 26 with the full sample (*N* = 321). The model demonstrated adequate fit [χ^2^ = 222.577 (*df* = 124, *p* < 0.001); *RMSEA* = 0.050; *RMR* = 0.025; *GFI* = 0.932; *CFI* = 0.971; *NFI* = 0.971], with no critical multicollinearity issues As shown in Table 4 and Figure 2, all three P-ESG dimensions significantly influenced *brand trust*: perceived environmental (β = 0.382, *t* = 4.807^*^), social perception (β = 0.248, *t* = 3.636^***^), and perceived governance (β = 0.219, *t* = 3.233^**^), fully supporting H1b, H2b and H3b. Perceived environmental (β = 0.205, *t* = 2.435^*^) and perceived governance (β = 0.328, *t* = 4.308^***^) positively predicted *brand identification*, validating H1a and H3a, whereas perceived social's effect (β = 0.144, *t* = 1.944, *p* > 0.05) rejected H2a. Moreover, b*rand identification* (β = 0.426, *t* = 6.371^***^) and *brand trust* (β = 0.344, *t* = 5.229^***^) each significantly enhanced *LCPI*, strongly supporting H4 and H5. At last, all control variables (e.g., gender, age, education and monthly disposable income) exhibited no substantive impact on the model.

**Table 4:** Structural analysis results (*N* = 321).

**Pathway**		**S. Estimate**	**S. E**.	***T***	**Results**
H1a	Perceived environmental → brand identification	0.205	0.069	2.435^*^	Accepted
H2a	Perceived society → brand identification	0.144	0.063	1.944	Rejected
H3a	Perceived governance → brand identification	0.328	0.061	4.308^***^	Accepted
H1b	Perceived environmental → brand trust	0.380	0.062	4.807^***^	Accepted
H2b	Perceived society → brand trust	0.248	0.056	3.636^***^	Accepted
H3b	Perceived governance → brand trust	0.219	0.052	3.233^**^	Accepted
H4	Brand identification → LCPI	0.426	0.063	6.371^***^	Accepted
H5	Brand trust → LCPI	0.344	0.064	5.229^***^	Accepted

Model fit summary: χ2(124) = 222.577; χ2/df =1.759; GFI = 0.932; CFI = 0.971; NFI = 0.937; RMSEA = 0.050; RMR = 0.025; ^*^p < 0.05; ^**^p < 0.01; ^***^p < 0.001. Sources: table by authors.

### 4.4 Moderating effect of consumer's green skepticism

**Figure 3:**
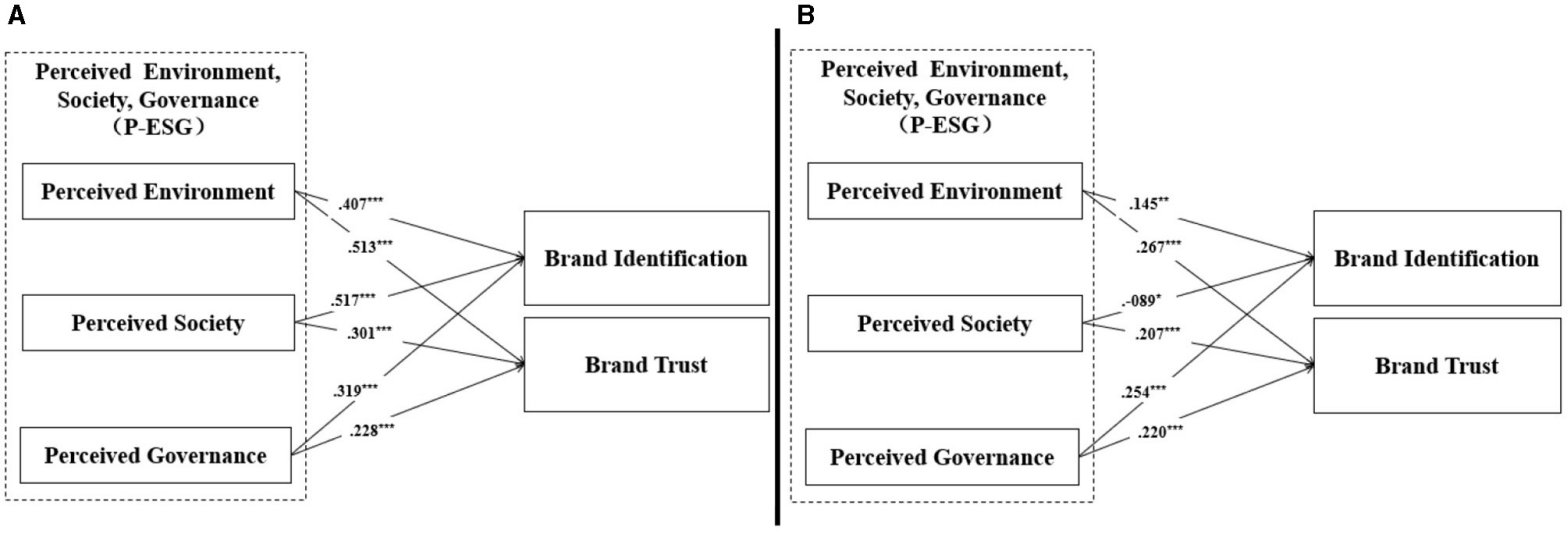
Results of structural analysis for different green skepticism groups. **(A)** Low-level green skepticism group. **(B)** High-level green skepticism group. Sources: figure by authors. *p < 0.05; **p < 0.01; ***p < 0.001.

In order to examine H6's prediction that P-ESG effects on brand identification and trust differ across consumers' different green skepticism levels, considering the sample size of less than 500, this study following methodological precedent (Luan et al., 2023; Wang et al., 2022) conducted multi-group SEM analysis by median-splitting the sample into low-level (*n*_1_ = 166) and high-level (*n*_2_ = 155) green skepticism groups based on participants' skepticism scores. For low-level green skepticism participants, all three P-ESG dimensions demonstrated significant positive impacts on both brand identification and trust, that is, perceived environmental (β_1_ = 0.407, *t*_1_ = 4.951^***^; β_2_ = 0.513, *t*_2_ = 5.114^***^), perceived social (β_1_ = 0.517, t_1_ = 6.417^***^; β_2_ = 0.301, *t*_2_ = 4.710^***^), and perceived governance (β_1_ = 0.319, *t*_1_ = 4.928^***^; β_2_ = 0.228, *t*_2_ = 3.998^***^). Furthermore, in the high-level green skepticism group, perceived environmental (β = 0.145, *t* = 3.182^**^) and governance perceptions (β = 0.254, *t* = 3.912^***^) maintained positive effects on brand identification, and all P-ESG dimensions had positive effects on brand trust. While, perceived social cues unexpectedly exhibited a negative relationship with brand identification (β = −0.089, *t* = −2.103^*^). Comparative analysis revealed that all path coefficients in the low-level green skepticism group exceeded those in the high-level green skepticism group, partially supporting H6 (Hair et al., 2011; Table 5, Figure 3).

**Table 5:** Structural analysis for two separate group.

**Pathway**	**Low-level green skepticism (Group A**, ***n***_**1**_ = **166)**		**High-level green skepticism (Group B**, ***n***_**2**_ = **155)**	
	**Path coefficient**	***t***	**Path coefficient**	***t***
Perceived environmental → brand identification	0.407	4.951^***^	0.145	3.182^**^
Perceived society → brand identification	0.517	6.417^***^	−0.089	−2.103^*^
Perceived governance → brand identification	0.319	4.928^***^	0.254	3.912^***^
Perceived environmental → brand trust	0.513	5.114^***^	0.267	4.540^***^
Perceived society → brand trust	0.301	4.710^***^	0.207	3.648^***^
Perceived governance → brand trust	0.228	3.998^***^	0.220	3.920^***^

^*^p < 0.05; ^**^p < 0.01; ^***^p < 0.001. Sources: table by authors.

Finally, to check the moderating role of consumers' green skepticism types on P-ESG → brand identification and brand trust, Δχ*2* (*df* = 1) value between free and constraint models in each path were calculated. The results revealed that there were significant differences at the α = 0.05 level for three of the paths: (PE → BI: Δχ*2* = 5.0 > 3.84; PS → BI: Δχ*2* = 44.1 > 3.84; PE → BT: Δχ^2^ = 4.1 > 3.84). Whereas, no significant differences were found in “PG → BI: Δχ^2^ = 0.3 <3.84; PS → BT: Δχ^2^ = 1.0 <3.84; PG → BT: Δχ^2^ = 0.1 <3.84” paths. Thus, H6 was partially supported (see Table 6).

**Table 6:** Moderating role of green skepticism.

**Pathway**		***χ^**2**^***		**Δ*χ^2^*** **(*df* = 1)**	***p*-value**	**Results**
		**Free model**	**Constraint model**			
H6a	Perceived environmental → brand identification	772.1 (*df* = 172, *p* < 0.001)	777.1	5.0	<0.05	Accepted
H6b	Perceived society → brand identification		816.2	44.1	<0.05	Accepted
H6c	Perceived governance → brand identification		772.4	0.3	n.s.	Rejected
H6d	Perceived environmental → brand trust		776.2	4.1	<0.05	Accepted
H6e	Perceived society → brand trust		773.1	1.0	n.s.	Rejected
H6f	Perceived governance → brand trust		772.2	0.1	n.s.	Rejected

Standard Δχ^2^(df = 1) = 3.84; n.s., not supported. Sources: table by authors.

## 5 Conclusion, discussion and limitation

### 5.1 Conclusion

The study investigates the relationship between “P-ESG, brand identification/brand trust, and consumers' LCPI.” Additionally, it also investigates the moderating role of consumers' green skepticism type on the relationships between P-ESG's all three dimensions and the constructs of brand attitude. The results indicate that all three P-ESG perceptions—environmental, social, and governance—significantly enhance consumers' brand trust. Moreover, perceived environmental and governance have a positive effect on brand identification, thereby supporting hypotheses H1a, H3a, H1b, H2b and H3b. Whereas, the significant effect of perceived society on brand identification is not observed, thus rejecting H2a. Consistent with expectations, brand identification and trust have significant positive effects on consumers' LCPI, which supports hypotheses H4 and H5.

Additionally, the results further indicate that in low-level (vs. high-level) green skepticism participant group, perceived environmental has significantly stronger effects on both brand identification and brand trust, moreover, perceived society has a stronger impact on brand identification. Whereas, there are no significant differences in “perceived governance → brand identification,” “perceived society → brand trust” and “perceived governance → brand trust” relationships during both high and low-level green skepticism participant groups. Thus, H6 was partially supported.

### 5.2 Theoretical implications

Firstly, this study advances signaling theory by operationalizing its principles within firm's ESG communication frameworks and sustainable consumption paradigms, decoding how consumers process P-ESG cues during low-carbon consumption decision-making. Three contributions emerge: (1) This study establishes a hierarchical efficacy of P-ESG dimensions (including perceived environmental, social and governance) in predicting consumers' brand trust, revealing environmental perceptions as the dominant signal in trust formation. (2) It inverts this hierarchy for brand identification outcomes, with governance, surpassing environmental and social cues, suggesting institutional credibility signals outweigh ecological ones in brand identification (Li, 2023). Moreover, the signaling theory highlights the importance of signal cost and authenticity, offering a valuable lens to distinguish between superficial ESG claims and substantive corporate actions. This distinction is essential in understanding which ESG initiatives most effectively foster consumers' brand evaluation (Konuk, 2023).

Secondly, extending prior investigations on ESG communication strategies (Wang S. et al., 2021), this study highlights the critical yet understudied roles of brand identification and brand trust in enhancing consumers' LCPI. Current research on ESG and sustainable consumption has largely overlooked these psychological mechanisms that drive eco-conscious purchasing decisions (Huang et al., 2024; Khalil and Khalil, 2022). Compare to Chen et al. (2023)'s one-dimensional insights on the relationship between governance transparency and consumer uncertainty, through theoretical expansion of signaling frameworks, this study demonstrates how corporate branding strategies exert profound influence on consumer behavior within emerging low-carbon markets. This approach addresses recent academic appeals for investigating behavioral catalysts in environmental consumption, particularly regarding the implementation of ESG communication strategies to narrow the attitude-behavior gap (Boufounou et al., 2023; Yu et al., 2025).

Thirdly, current research demonstrates limited attention to divergent low-carbon consumption patterns among consumers with varying skepticism levels regarding firm's environmental claims (Luo et al., 2020). The findings reveal that green skepticism partially moderates the pathways connecting P-ESG with brand identification and brand trust, notably demonstrating that governance- and society-related ESG dimensions show no significant behavioral differentiation across different skepticism groups. This investigation advances low-carbon consumption research through three critical expansions: (1) integrating communication strategy with green value theory, (2) establishing consumer skepticism as a behavioral boundary condition, and (3) providing improved explanatory capacity for nuanced behavioral variations. The non-significant governance/society effects may stem from institutional familiarity - as global sustainability awareness, consumers develop cognitive schemas that prioritize holistic ESG evaluations over discrete governance/society analysis (Chen, 2020). This information-processing heuristic enables brand perception formation without meticulous scrutiny of specific institutional or social responsibility factors (Wang et al., 2024b).

### 5.3 Managerial implications

This study advances the conceptualization of sustainable consumption dynamics by elucidating how multidimensional perceptual constructs in ESG strategies activate consumer intentions toward low-carbon products through brand attitude mechanisms. The findings provide strategic frameworks for optimizing firm's ESG communication architectures, and informing policy development for environmental governance systems. These dual contributions bridge critical gaps between organizational sustainability practices and societal decarbonization imperatives.

Specifically, the analytical insights reveal three strategic priorities for firm's ESG communication implementation. First, environmental stewardship and governance rigor emerge as critical drivers, with consumers' perceptions of these dimensions significantly strengthening brand connections, necessitating concrete operationalization through phased decarbonization roadmaps, for instance, transitioning manufacturing facilities to solar power, and paired with governance innovations like publishing board diversity reports and linking 30% of executive bonuses to independently verified sustainability KPIs. Second, while perceived social dimensions show limited impact on brand identity formation, their trust-building potential requires bifurcated social strategies, that is, maintaining baseline compliance through certifications, such as Fair Wage Coalition accreditation. Moreover, such as urban green spaces with QR codes, the firms should simultaneously sponsor high-visibility initiatives to transform abstract social claims into tangible social proof. Third, the robust brand-LCPI linkage demands operationalized trust mechanisms extending beyond conventional quality improvements; this includes developing blockchain-enabled carbon traceability systems allowing real-time supply chain verification, such as “IBM Food Trust for supply chain audits” implementations. The firms could couple with post-purchase impact reporting via automated monthly emails detailing contribution metrics to reforestation initiatives, such as “Your purchase protected 3 m^2^ of xx forest,” to reinforce consumers' behavioral reinforcement loops (Yoshida et al., 2021; Gurler, 2025).

Moreover, this study identifies consumers' green skepticism as a pivotal moderating factor that shapes the translation of P-ESG into consumers' brand identification and trust. Low-level skepticism consumers exhibit stronger brand alignment through perceived environmental and social dimensions via value-congruence mechanisms, such as associating sustainable practices with personal health benefits or cost savings. For instance, strategies exemplified by Unilever's “Clean Future” campaign strategically link plant-based ingredients to skin health improvements. Conversely, high- level skepticism consumers require forensic verification protocols to overcome cognitive discounting, necessitating multi-layered evidence systems like “IBM Food Trust” for blockchain-enabled carbon traceability combined with satellite-imagery deforestation monitoring (e.g., Nestlé's Reforestation Tracker). To bridge this divide, the research advocates for segmented ESG communication strategies aligned with consumers' varying skepticism levels. For low-level green skepticism consumers, the study recommends a dual-focused communication approach that integrates firm's sustainability initiatives with tangible consumer benefits – including health improvements and cost-efficiency gains – to strengthen engagement through perceived value alignment. In contrast, high-skepticism audiences require evidence-based persuasion strategies. Symbolic ESG claims prove insufficient for this group, necessitating multifaceted verification systems such as third-party certifications (e.g., CDP ratings), transparent carbon accounting methodologies (including Scope 3 emissions), and demonstrable societal impact metrics (e.g., community reinvestment ratios or verifiable emission-reduction milestones). These evidence hierarchies effectively address their heightened verification demands.

Finally, the research further emphasizes the importance of establishing sustainable credibility architectures. Operationally, firms should prioritize auditable sustainability practices, including renewable energy adoption rates and circular economy implementations (e.g., product lifecycle assessments), to build credibility reservoirs. Communicatively, the study suggests reframing low-carbon consumption from individual obligation narratives to collective value co-creation paradigms. Innovative tactics such as real-time environmental performance dashboards and expert endorsements (Worakittikul et al., 2024) could recalibrate skeptical consumers' cost-benefit calculus, ultimately bridging the perception-attitude gap through enhanced brand identification and trust calibration mechanisms.

### 5.4 Limitations

This study presents four notable limitations that delineate directions for future research. Firstly, the data collection methodology lacked product specificity, potentially introducing preference-related confounding effects. Future experimental designs should incorporate cross-category product sampling to control for preference-driven biases and strengthen ecological validity. Secondly, while the developed framework integrates firm's ESG performance with consumer psychographics, it partially addresses the multidimensional nature of low-carbon decision-making. Subsequent research should expand the analytical scope to include: (1) digital engagement tactics (e.g., AI-driven personalization), (2) regulatory landscapes (e.g., carbon credit mechanisms), and (3) organizational intervention strategies. Such extensions would elucidate boundary conditions and operational complexities in sustainable consumption ecosystems. Thirdly, as Chinese people's awareness of ESG is increasing, the exclusive focus on Chinese consumers seems reasonable from a certain perspective. While, it limits the cross-cultural generalizability. Future study should validate these mechanisms in culturally diverse markets (e.g., individualistic vs. collectivist societies) to establish universal boundary conditions for ESG-driven brand attitude formation. Fourthly, the survey methodology requiring participants to recall a single brand before completing the questionnaire may overlook the potential influence of brand attributes and market size. To enhance the robustness of future investigations, these factors should be incorporated as control variables or moderating variables in subsequent study.

## Data Availability

The raw data supporting the conclusions of this article will be made available by the authors, without undue reservation.

## Appendix

**Table A1:** List of scale items.

**Variable**		**Item**	**Source**
Perceived Environmental, Social, Governance (P-ESG)	Perceived environmental	(PE1) The company establishes and implements policies to reduce carbon emissions.	Oh et al., 2024
		(PE2) The company engages in various actions and social campaigns to protect the environment.	
		(PE3) The company demonstrates proactive alignment with international sustainability certifications by integrating recognized environmental protocols into core business operations.	
	Perceived society	(PS1) The company ensures stable employment and a positive working environment.	
		(PS2) The company provides good products and services and constantly strives to improve its product quality.	
		(PS3) The company is well-equipped with consumer remedy systems.	
	Perceived governance	(PG1) The company's ownership/governance structure is stable and transparent.	
		(PG2) The company has a transparent system of internal audits.	
		(PG3) The employees comply with workplace ethics and anti-corruption guidelines.	
Brand trust		(BT1) This company delivers what it promises.	Hur et al., 2014
		(BT2) This company's product claims are believable.	
		(BT3) This company has a name you can trust.	
Brand identification		(BI1) My personality is very similar to the personality of this brand.	Tuškej et al., 2013
		(BI2) I have a lot in common with other people who use this brand.	
		(BI3) My values are very similar to the values of this brand.	
Green skepticism		(GS1) Most information of low-carbon product on social media are exaggerated or false.	Luo et al., 2020; Yu, 2020
		(GS2) Most communication messages about low-carbon product are intended to deceive rather than inform.	
		(GS3) Most advertisements of low-carbon product aim to get more profits.	
Control variable	Environmental preference	(EP1) I am willing to buy biodegradable packaging products.	Shimul and Cheah, 2023
		(EP2) Even give up some function or shopping experience, I will also buy eco-friendly products.	
		(EP3) I am willing to replace products for environmental reasons.	
	Perceived environmental premium	(PEP1) I am willing to pay more for much more eco-friendly materials.	Shi and Jiang, 2023
		(PEP2) Although the price of eco-friendly materials is more expensive, it has the value of buying them.	
		(PEP3) I am willing to pay more for customizing much more eco-friendly materials.	
Low-carbon Purchase Intention (LCPI)		(LCPI1) I will consider purchasing low-carbon products of this company.	Wang et al., 2023
		(LCPI2) I am willing to buy low-carbon products of this corporate.	
		(LCPI3) I will buy low-carbon products of this corporate within 6 months.	
